# Efficacy of M_split_ Estimation in Displacement Analysis

**DOI:** 10.3390/s19225047

**Published:** 2019-11-19

**Authors:** Zbigniew Wiśniewski, Robert Duchnowski, Andrzej Dumalski

**Affiliations:** Institute of Geodesy, University of Warmia and Mazury in Olsztyn, 1 Oczapowskiego St., 10-957 Olsztyn, Poland; zbyszekw@uwm.edu.pl (Z.W.); andrzej.dumalski@uwm.edu.pl (A.D.)

**Keywords:** M_split_ estimation, efficacy, Monte Carlo simulations, deformation analysis

## Abstract

Sets of geodetic observations often contain groups of observations that differ from each other in the functional model (or at least in the values of its parameters). Sets of observations obtained at various measurement epochs is a practical example in such a context. From the conventional point of view, for example, in the least squares estimation, subsets in question should be separated before the parameter estimation. Another option would be application of M_split_ estimation, which is based on a fundamental assumption that each observation is related to several competitive functional models. The optimal assignment of every observation to the respective functional model is automatic during the estimation process. Considering deformation analysis, each observation is assigned to several functional models, each of which is related to one measurement epoch. This paper focuses on the efficacy of the method in detecting point displacements. The research is based on example observation sets and the application of Monte Carlo simulations. The results were compared with the classical deformation analysis, which shows that the M_split_ estimation seems to be an interesting alternative for conventional methods. The most promising are results obtained for disordered observation sets where the M_split_ estimation reveals its natural advantage over the conventional approach.

## 1. Introduction and Motivation

Consider the classical functional model of geodetic observations, which is given for l=1,…,q different measurement epochs, namely

(1) $$y=AX+v\;\Rightarrow \;y_{l}=A_{l}X_{l}+v_{l}$$

where $y_{l}={\left[y_{1,l},\cdots ,y_{n_{l},l}\right]}^{T}$ are the observation vectors whose elements belong to the respective sets $\Phi_{l}=\{y_{1,l},\ldots ,y_{n_{l},l}\}$; $X_{l}={\left[X_{1,l},\cdots ,X_{r,l}\right]}^{T}$ are the parameter vectors; $v_{l}={\left[v_{1,l},\cdots ,v_{n_{l},l}\right]}^{T}$ are vectors of random errors; and $A_{l}\in R^{n_{l},r}$ are known coefficient matrices. Such models are the basis of deformation analysis, namely for determining the shifts $\Delta X_{\left(k,l\right)}=X_{l}-X_{k}$ between the epochs *l* and *k* (for example, the changes of the point coordinates between such epochs).

The vectors $\Delta X_{\left(k,l\right)}$ can be estimated by applying different methods or strategies (e.g., [1,2,3]). The least squares method (LS-method) is still the most popular approach in such an analysis, note that LS-estimates are often supplemented with respective statistical tests (e.g., [4,5,6]). However, some unconventional methods are also in use, for example, robust M-estimation [7,8] or R-estimation [9,10,11,12,13,14]. In the case of relative networks, one can also apply methods of free adjustment (e.g., [15,16,17,18]). Some methods as well as their properties are well known, other methods are still being researched.

The M_split_ estimation surely belongs in the latter group. The M_split_ estimation was proposed by Wiśniewski [19,20] and has been applied to some practical problems in which each observation could be assigned to several different functional models. For example, it was used in remote sensing (terrestrial laser scanning or ALS data) for data modeling [21] and in some geodetic problems, for example, in deformation analysis [22,23,24,25], and robust estimation (e.g., [26]). Automatic assignment of each observation to the best fitted model is one of the most important features of M_split_ estimation. It is also very useful in deformation analysis, when the observation set might include observations from all measurement epochs (the set is an unrecognized mixture of such observations). Note that there is usually no problem with separating observations from different epochs and hence with separate analyses. However, there are some cases when the application of the M_split_ estimation is advisable. For example, when a point is displaced during an observation session, thus, one should consider two pseudo-epochs, and the M_split_ estimation allows us to estimate the parameters of the functional models for such pseudo-epochs. Such models can also be applied when an observation set is disturbed by outliers [23,24]. Note that the method under investigation can be applied in all observation sets that are an unrecognized (and/or unordered) mixture of observation aggregations. Such data can result from different sources or instrumentations. In fact, the source of data does not matter here. It can be, for example, geodetic instruments: total stations, GNSS receivers, etc., or in remote sensing such as terrestrial or airborne laser scanners.

The main properties of the M_split_ estimation are discussed in the papers cited above; that study focused on the efficacy of the method in estimating parameters of the competitive functional models, hence also in estimating point displacements. The analyses were based on simulations of the crude Monte Carlo method and the application of elementary functional models or models of a leveling network. The results were compared with the results of the LS-method.

## 2. Theoretical Foundations

Without loss of generality, we can assume two measurement epochs, thus in the model of Equation (1), we have q=2. Then, the optimization criterion of the LS-method and its solution can be written in the following way (l=1,2)

(2) $$\varphi_{LS}(X_{l})=\sum_{i=1}^{n}v_{i,l}^{2}p_{i,l}=v_{l}^{T}P_{l}v_{l}=\min\;\to \;{\hat{X}}_{LS,l}=D_{LS,l}y_{l}$$

where $v_{i,l}=y_{i,l}-a_{i,l}X_{l}$, $D_{LS,l}={\left(A_{l}^{T}P_{l}A_{l}\right)}^{-1}A_{l}^{T}P_{l}$, $P_{l}$ are respective weight matrices, ($a_{i,l}$– *i*th row of matrix $A_{\left(l\right)}$). The difference $\Delta {\hat{X}}_{LS(1,2)}={\hat{X}}_{LS,2}-{\hat{X}}_{LS,1}$ is a LS-estimate of the shift $\Delta X_{\left(1,2\right)}$. 

In the case of the M_split_ estimation, we assumed that each observation belonged to either of two sets $\Phi_{1}$ or $\Phi_{2}$; however, there is one observation set $\Phi =\Phi_{1}\cup \Phi_{2}$ and one observation vector $y={\left[y_{1},\cdots ,y_{n}\right]}^{T}$, $n=n_{1}+n_{2}$. There are two competitive functional models

(3) $$y=AX_{\left(1\right)}+v_{\left(1\right)},\;y=AX_{\left(2\right)}+v_{\left(2\right)}$$

with two competitive versions of the parameter X, namely $X_{\left(1\right)}$ and $X_{\left(2\right)}$ ($A\in R^{n,r}$,rank(A)=r). The vectors $v_{\left(1\right)},v_{\left(2\right)}\in R^{n}$ are two competitive versions of the observation errors related to all elements of the vector **y**.

The theoretical basis of the M_split_ estimation is an assumption that every observation $y_{i}$ can be assigned to either of two density function $f(y_{i};X_{\left(1\right)})$ or $f(y_{i};X_{\left(2\right)})$. If $y_{i}$ occurs, it brings the *f*-information $I_{f}(y_{i};X_{\left(1\right)})=-\ln f(y_{i};X_{\left(1\right)})$ or the *f*-information $I_{f}(y_{i};X_{\left(2\right)})=-\ln f(y_{i};X_{\left(2\right)})$, which are competitive to each other. M_split_ estimates of the parameters $X_{\left(1\right)}$ and $X_{\left(2\right)}$, namely ${\hat{X}}_{\left(1\right)}$ and ${\hat{X}}_{\left(2\right)}$, minimize the following global information that is brought by all elements of the vector y [19]

(4) $$I_{f}(y;X_{\left(1\right)},X_{\left(2\right)})=\sum_{i=1}^{n}I_{f}(y_{i};X_{\left(1\right)})I_{f}(y_{i};X_{\left(2\right)})=\sum_{i=1}^{n}\left[-\ln f(y_{i};X_{\left(1\right)})][-\ln f(y_{i};X_{\left(2\right)})\right]$$

In other words, the estimators in question are the solutions of the following optimization problem:

(5) $$\min I_{f}(y;X_{\left(1\right)},X_{\left(2\right)})=\min I_{f}(y;{\hat{X}}_{\left(1\right)},{\hat{X}}_{\left(2\right)})$$

For such solutions, the occurrence of the particular observation vector is the most probable. If $X_{\left(1\right)}=X_{\left(2\right)}=X$, then $I_{f}(y;X_{\left(1\right)},X_{\left(2\right)})=\varphi_{ML}(X)=\sum_{i=1}^{n}\left[-\ln f(y_{i};X)\right]$, which is the objective function of the maximum likelihood method (ML-method). In such a context, the M_split_ estimation is a special development of the ML-method. Huber [27,28] generalized the ML-method to M-estimation by introducing $\varphi_{M}(X)=\sum_{i=1}^{n}\rho (y_{i};X)$, where $\rho (y_{i};X)$ is an arbitrary function for which estimators obtain the desired properties (for example, they are robust against outliers). A similar generalization was also proposed for the M_split_ estimation [19,20]. The objective function of Equation (4) is replaced by the following function

(6) $$\varphi_{\rho }(X_{\left(1\right)},X_{\left(2\right)})=\sum_{i=1}^{n}\rho_{\left(1\right)}(y_{i};X_{\left(1\right)})\rho_{\left(2\right)}(y_{i};X_{\left(2\right)})$$

Of course, M_split_ estimation is also a development of classical M-estimation, if only $X_{\left(1\right)}=X_{\left(2\right)}=X$, and hence $\varphi_{\rho }(X_{\left(1\right)},X_{\left(2\right)})=\varphi_{M}(X)$.

There are several variants of M_split_ estimation that differ from one another in the objective function or assumed parameters [19,22,29]. So far, the most popular is the squared M_split_ estimation for which $\rho_{\left(1\right)}(y_{i};X_{\left(1\right)})=p_{i}v_{i(1)}^{2}$ and $\rho_{\left(2\right)}(y_{i};X_{\left(2\right)})=p_{i}v_{i(2)}^{2}$. Hence, one can write the following optimization problem of such a method [19,30] as

(7) $$\varphi_{sq}(X_{\left(1\right)},X_{\left(2\right)})=\sum_{i=1}^{n}p_{i}^{2}v_{i(1)}^{2}v_{i(2)}^{2}={\left(v_{\left(1\right)}*v_{\left(1\right)}\right)}^{T}P^{2}(v_{\left(2\right)}*v_{\left(2\right)})=\min$$

where $P=\mathrm{Diag}(p_{1},\ldots ,p_{n})$ is a diagonal weight matrix of the observations y (∗ – the Hadamard product). It is obvious that if $X_{\left(1\right)}=X_{\left(2\right)}=X$, then $\varphi_{sq}(X_{\left(1\right)},X_{\left(2\right)})=\sum_{i=1}^{n}p_{i}v_{i}^{2}$, which means that the squared M_split_ estimation is a development of the LS method. Considering such a relationship and the range of the practical applications of the M_split_ estimation, we will only discuss the squared M_split_ estimation. To compute M_split_ estimates, one can use the sufficient conditions for the minimum of the objective function. Considering the optimization problem (7), one can write the following equations

$$\begin{array}{c} g_{\left(1\right)}(X_{\left(1\right)},X_{\left(2\right)})={\left(\partial \varphi (X_{\left(1\right)},X_{\left(2\right)})/\partial X_{\left(1\right)}\right)}_{\begin{array}{l} X_{\left(1\right)}={\hat{X}}_{\left(1\right)}\\ X_{\left(2\right)}={\hat{X}}_{\left(2\right)} \end{array}}^{T}=0\\ g_{\left(2\right)}(X_{\left(1\right)},X_{\left(2\right)})={\left(\partial \varphi (X_{\left(1\right)},X_{\left(2\right)})/\partial X_{\left(2\right)}\right)}_{\begin{array}{l} X_{\left(1\right)}={\hat{X}}_{\left(1\right)}\\ X_{\left(2\right)}={\hat{X}}_{\left(2\right)} \end{array}}^{T}=0 \end{array}\Leftrightarrow$$

(8) $$\begin{array}{c} A^{T}w_{\left(1\right)}({\hat{v}}_{\left(2\right)}){\hat{v}}_{\left(1\right)}=A^{T}w_{\left(1\right)}({\hat{v}}_{\left(2\right)})(y-A{\hat{X}}_{\left(1\right)})=0\\ A^{T}w_{\left(2\right)}({\hat{v}}_{\left(1\right)}){\hat{v}}_{\left(2\right)}=A^{T}w_{\left(2\right)}({\hat{v}}_{\left(1\right)})(y-A{\hat{X}}_{\left(2\right)})=0 \end{array}$$

where $g_{\left(1\right)}(X_{\left(1\right)},X_{\left(2\right)})$ and $g_{\left(2\right)}(X_{\left(1\right)},X_{\left(2\right)})$ are the gradients of the function $\varphi_{sq}(X_{\left(1\right)},X_{\left(2\right)})$. The following matrices

(9) $$w_{\left(1\right)}(v_{\left(2\right)})=\mathrm{Diag}\left(\ldots ,w_{\left(1\right)}(v_{i(2)}),\ldots \right),\;w_{\left(2\right)}(v_{\left(1\right)})=\mathrm{Diag}\left(\ldots ,w_{\left(2\right)}(v_{i(1)}),\ldots \right)$$

are diagonal weight matrices that are based on the cross-weighting functions [20,31]

(10) $$w_{\left(1\right)}(v_{i(2)})=\frac{\partial p_{i}^{2}v_{i(1)}^{2}v_{i(2)}^{2}}{2v_{i(1)}\partial v_{i(1)}}=p_{i}^{2}v_{i(2)}^{2},\;w_{\left(2\right)}(v_{i(1)})=\frac{\partial p_{i}^{2}v_{i(1)}^{2}v_{i(2)}^{2}}{2v_{i(2)}\partial v_{i(2)}}=p_{i}^{2}v_{i(1)}^{2}$$

The solutions of Equation (8) are the following M_split_ estimators

(11) $${\hat{X}}_{\left(1\right)}=D_{\left(1\right)}({\hat{v}}_{\left(2\right)})y,\;{\hat{X}}_{\left(2\right)}=D_{\left(2\right)}({\hat{v}}_{\left(1\right)})y$$

where

(12) $$D_{\left(1\right)}({\hat{v}}_{\left(2\right)})={\left[A^{T}w_{\left(1\right)}({\hat{v}}_{\left(2\right)})A\right]}^{-1}A^{T}w_{\left(1\right)}({\hat{v}}_{\left(2\right)}),\;D_{\left(2\right)}({\hat{v}}_{\left(1\right)})={\left[A^{T}w_{\left(2\right)}({\hat{v}}_{\left(1\right)})A\right]}^{-1}A^{T}w_{\left(2\right)}({\hat{v}}_{\left(1\right)})$$

Thus, ${\hat{X}}_{\left(1\right)}$ is a function of ${\hat{v}}_{\left(2\right)}=y-A{\hat{X}}_{\left(2\right)}$, whereas ${\hat{X}}_{\left(2\right)}$ is a function of ${\hat{v}}_{\left(1\right)}=y-A{\hat{X}}_{\left(1\right)}$. For this reason, this solution has an asymptotic character. The following iterative procedure can be applied to compute the sought estimates (j=1,…,m)

(13) $$\begin{array}{l} X_{\left(1\right)}^{j+1}=D_{\left(1\right)}(v_{\left(2\right)}^{j})y,\;v_{\left(1\right)}^{j+1}=y-AX_{\left(1\right)}^{j+1}\\ X_{\left(2\right)}^{j+1}=D_{\left(2\right)}(v_{\left(1\right)}^{j+1})y,\;v_{\left(2\right)}^{j+1}=y-AX_{\left(2\right)}^{j+1}\; \end{array}$$

(for the given starting point, for example, $v_{\left(2\right)}^{0}=y-A{\hat{X}}_{LS}$). The process stops when for each l=1,2, it holds that $g_{\left(l\right)}({\hat{X}}_{\left(1\right)},{\hat{X}}_{\left(2\right)})=0$ and hence ${\hat{X}}_{\left(l\right)}=X_{\left(l\right)}^{m}=X_{\left(l\right)}^{m-1}$. Note that other iterative processes that use both the gradients and the Hessians of $\varphi (X_{\left(1\right)},X_{\left(2\right)})$, namely Newton’s method, can be found in [19,20,29].

**Figure 1:**
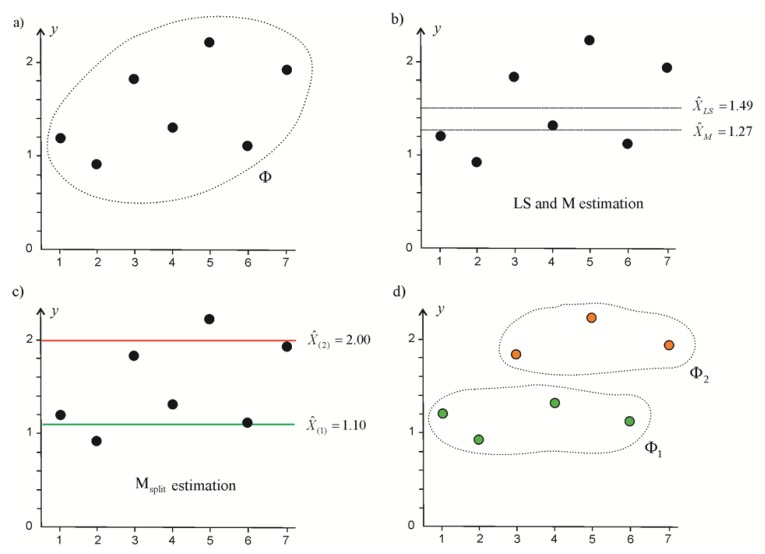
Least squares, robust M-estimate and both M_split_ estimates within a sample observation set (**a**) observation set; (**b**) classical estimates; (**c**) M_split_ estimates; (**d**) observation subsets.

Now, the elementary property the of M_split_ estimates is shown. Here, we consider a basic example that precedes the more detail analysis presented in the next section. Let us assume the functional model $y_{i}=X+v_{i}$, i=1,…,7, and the observation set Φ as a following vector $y=\begin{array}{ccccccc} {}[1.2 & 0.9 & 1.8 & 1.3 & 2.2 & 1.1 & 1.9{]}^{T} \end{array}$ (Figure 1a). Then, ${\hat{X}}_{LS}=1.49$. For the sake of comparison, let the robust M-estimate be computed. By applying the Huber method [27,28], where the weight function is w(v)=min{1, k/|v|} and k=3, one can obtain ${\hat{X}}_{M}=1.27$ (Figure 1b). Both estimates in question are not satisfactory, and do not reflect the nature of the observation set. The robust estimate ${\hat{X}}_{M}$ lies closer to the “bigger” aggregation of observations. Next, the question of how to treat the observations that are furthest from that estimate arises. In the classical approach, such observations are regarded as outliers (for example, affected by gross errors), and we are no longer interested in such observations. Different conclusions follow the M_split_ estimation where ${\hat{X}}_{\left(1\right)}=1.10$ and ${\hat{X}}_{\left(2\right)}=2.00$ (Figure 1c).

The M_split_ estimates show that set Φ consists of two subsets $\Phi_{1}$ and $\Phi_{2}$ (Figure 1d), whose elements can be regarded as realizations of two different random variables that differ from each other in location parameters $X_{1}$ and $X_{2}$, respectively. Similar assumptions can also be found in other estimation problems, for example, cluster analysis (e.g., [32,33]); or in a mixed model estimation applied in geosciences (e.g., [34,35]). Such approaches can be regarded as alternatives; however, we should have some understanding that they differ significantly in their general ideas. 

Assigning each observation to the model that is the most suitable for it is a natural process in M_split_ estimation. This property can be applied in the analysis of network deformation where there are two functional models:$\;y_{1}=A_{1}X_{1}+v_{1}$ and $\;y_{2}=A_{2}X_{2}+v_{2}$ for two measurement epochs, respectively. Thus, one can create one common observation vector $y={\left[y_{1}^{T},y_{2}^{T}\right]}^{T}$, the common weight matrix $P=\mathrm{Diag}\left(P_{\left(1\right)},P_{\left(2\right)}\right)$, and the coefficient matrix $A={\left[A_{1}^{T},A_{2}^{T}\right]}^{T}$. It is noteworthy that the order of the observation within vector y can be arbitrary. The actual order of the observations must coincide with the order of the rows within matrix A and order of the weights in weight matrix P. Here, the shift $\Delta X_{\left(1,2\right)}$ can be estimated by $\Delta {\hat{X}}_{\left(1,2\right)}={\hat{X}}_{\left(2\right)}-{\hat{X}}_{\left(1\right)}$. It is worth noting that $\Delta X_{\left(1,2\right)}$ can also be estimated directly by applying the Shift-M_split_ estimation proposed by Duchnowski and Wiśniewski [22].

## 3. Empirical Analyses

### 3.1. Elementary Tests

The elementary analysis was based on the univariate models and simulations of observations related to such models. Thus,

(14) $$\begin{array}{l} y_{i,1}=X_{1}+v_{i,1},\;i=1,\ldots ,n_{1}\\ y_{i,2}=X_{2}+v_{i,2},\;i=1,\ldots ,n_{2} \end{array}\Leftrightarrow \begin{array}{l} y_{1}=1_{n_{1}}X_{1}+v_{1}\\ y_{2}=1_{n_{2}}X_{2}+v_{2} \end{array}$$

where $1_{n_{l}}={\left[1_{1},\cdots ,1_{n_{l}}\right]}^{T}$; $X_{1}$ and $X_{2}$ are parameters that differ from each other in the shift $\Delta X_{\left(1,2\right)}=X_{2}-X_{1}$. The measurements, namely the elements of vectors $y_{1}$ and $y_{2}$, were simulated by using the Gaussian generator randn(n,1) of MATLAB. We assumed that σ=1, and the following theoretical values of the parameters: $X_{1}^{t}=0$ and hence $X_{2}^{t}=X_{1}^{t}+\Delta X_{\left(1,2\right)}=\Delta X_{\left(1,2\right)}$. Considering the LS-estimation of $X_{1}$ and $X_{2}$ we can apply the model of Equation (14) or Equation (1) where $A_{1}=1_{n_{1}}$ and $A_{2}=1_{n_{2}}$. In the case of M_split_ estimation, we assumed the model of Equation (3), taking $y={\left[y_{1}^{T},y_{2}^{T}\right]}^{T}\in R^{n}$, $n=n_{1}+n_{2}$, and $A={\left[1_{n_{1}}^{T},1_{n_{2}}^{T}\right]}^{T}=1_{n}$. We also applied the iterative procedure of Equation (13) by taking LS-estimates as the starting point (note that the starting point can usually be arbitrary).

**Figure 2:**
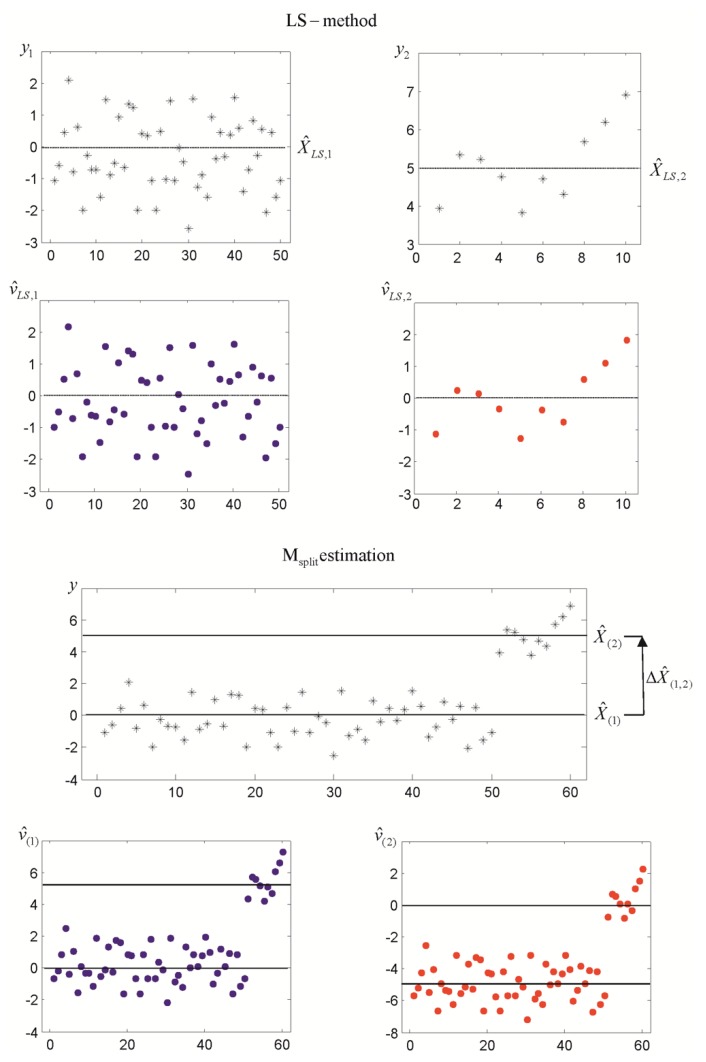
LS estimates, M_split_ estimates, and respective residuals (elementary functional models for $\Delta X_{\left(1,2\right)}=5$).

Let us now consider an example of observation simulation for which $\Delta X_{\left(1,2\right)}=5\sigma =5$ and $n_{1}=50$, $n_{2}=10$. The parameter estimates, together with the respective residuals, are presented in Figure 2.

**Figure 3:**
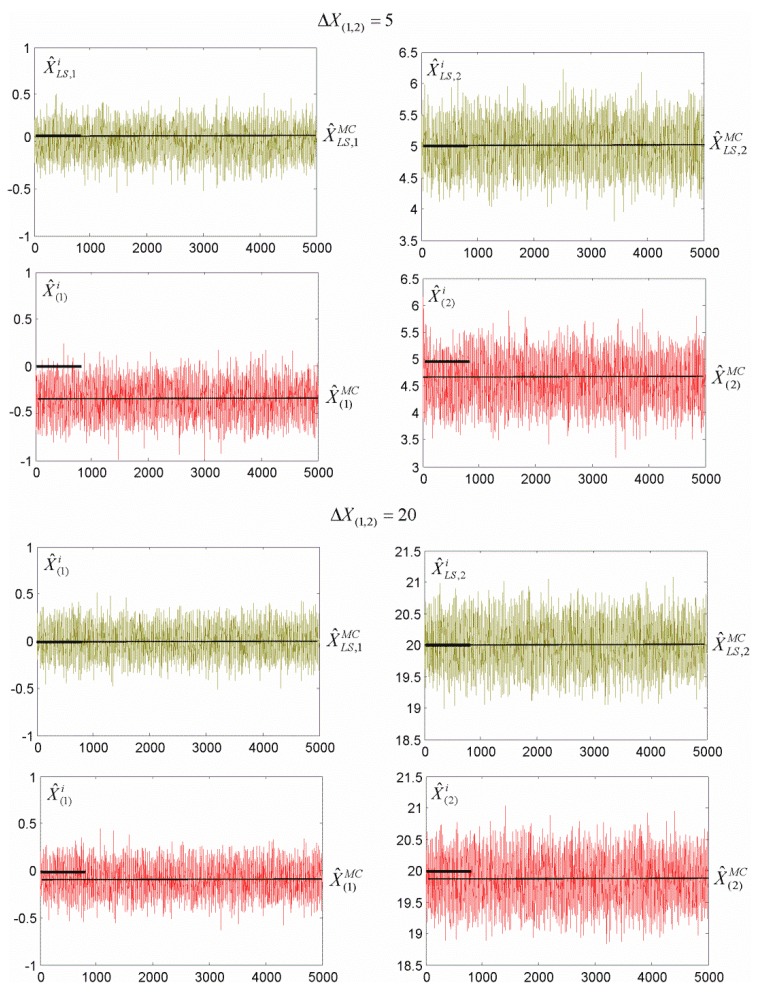
Location of the Monte Carlo estimates for $\Delta X_{\left(1,2\right)}=5\;$ or $\Delta X_{\left(1,2\right)}=20$ (for N=5000).

Now, let us consider more simulated observation sets. By applying the crude Monte Carlo method (MC) for *N* simulations, one can compute the MC estimates by applying the formula

(15) $${\hat{\theta }}_{}^{MC}=\frac{1}{N}\sum_{i=1}^{N}{\hat{\theta }}_{}^{i}$$

where ${\hat{\theta }}^{i}$ are the estimates obtained for the *i*th simulation. The location of the MC estimates for N=5000 and $\Delta X_{\left(1,2\right)}=5$ or $\Delta X_{\left(1,2\right)}=20$ is presented in Figure 3.

This shows that the MC estimates that were obtained for both estimation methods were close to the respective theoretical values (considering the simulated standard deviation). Generally, the LS estimates seemed more satisfactory. Please note that the results obtained for different values of shift $\Delta X_{\left(1,2\right)}$ indicate that M_split_ estimation is more satisfactory for bigger shifts than for smaller ones. Thus, let us examine how efficient the M_split_ estimation is for different shifts.

Let the measure of efficacy be defined in relation to the LS estimates, thus

(16) $$\lambda_{\left(l\right)}({\hat{X}}_{\left(l\right)},{\hat{X}}_{LS,l})=abs({\hat{X}}_{\left(l\right)}-X_{l}^{t})-abs({\hat{X}}_{LS,l}-X_{l}^{t})$$

Note that when $\lambda_{\left(l\right)}({\hat{X}}_{\left(l\right)},{\hat{X}}_{LS,l})<0$, then the M_split_ estimate is closer to the theoretical value than the LS estimate. Now, we can define the following function of an elementary success of M_split_ estimation

(17) $$s_{\left(l\right)}({\hat{X}}_{\left(l\right)},{\hat{X}}_{LS,l})=\{\begin{array}{c} 1\;for\;\lambda ({\hat{X}}_{\left(l\right)},{\hat{X}}_{LS,l})<0\\ 0\;for\;\lambda ({\hat{X}}_{\left(l\right)},{\hat{X}}_{LS,l})>0 \end{array}$$

The application of MC simulations allowed us to present the success rate (SR), which can be computed for different values of the shift $\Delta X_{\left(1,2\right)}$

(18) $$\gamma_{\left(l\right)}({\hat{X}}_{\left(l\right)},{\hat{X}}_{LS,l};\Delta X_{\left(1,2\right)})=\frac{1}{N}\sum_{i=1}^{N}s_{\left(l\right)}^{i}({\hat{X}}_{\left(l\right)},{\hat{X}}_{LS,l})$$

where $s_{\left(l\right)}^{i}({\hat{X}}_{\left(l\right)},{\hat{X}}_{LS,l})$ is the value of Equation (17) at the *i*th simulation.

**Figure 4:**
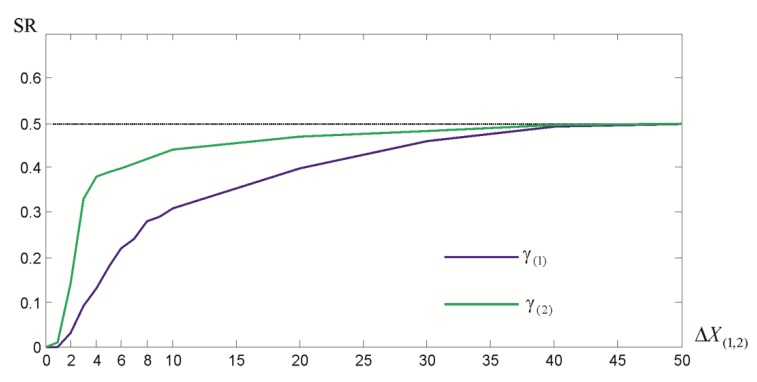
The success rates of M_split_ estimates ${\hat{X}}_{\left(1\right)}$ and ${\hat{X}}_{\left(2\right)}$ for the growing value of $\Delta X_{\left(1,2\right)}$.

Note that such a SR is defined in a very similar way to the mean success rate (MSR) given by Hekimoglu and Koch [36]. SRs for different $\Delta X_{\left(1,2\right)}$ and for N=5000 simulations are presented in Figure 4.

### 3.2. Vertical Displacement Analysis

**Figure 5:**
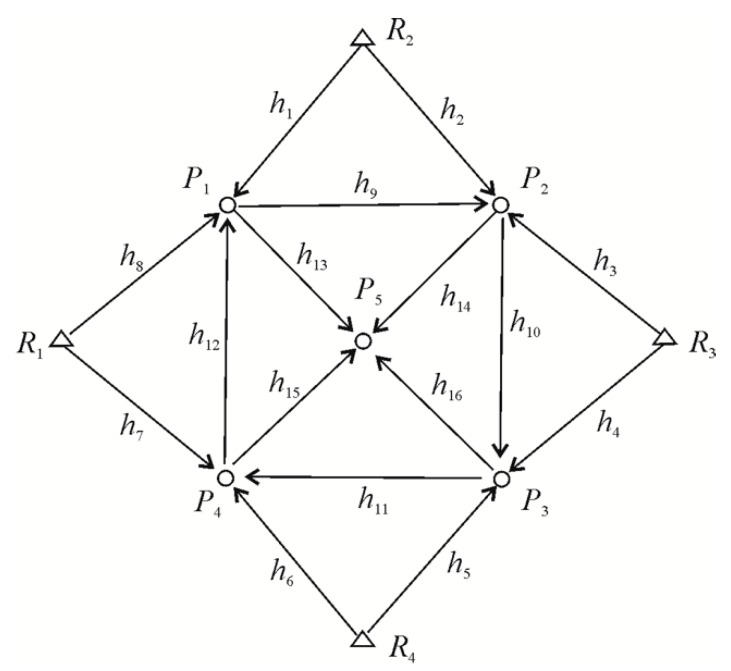
Tested leveling network.

Let us now consider the efficacy of M_split_ estimates in a more practical example, namely the analysis of vertical displacements within the leveling network, which is presented in Figure 5. Such a network has already been under investigation in previous papers [24,25]. 

The network consists of four reference points $R_{1},\ldots ,R_{4}$ with the known heights $H_{R_{1}}=\cdots =H_{R_{4}}=0$ m and five object points of $P_{1},\ldots ,P_{5}$. We assumed that each of the height differences $h_{1},\ldots ,h_{16}$ was measured twice at each of two measurement epochs, and that σ=2 mm was the known standard deviation of all measurements. We also assumed that at the first epoch $X_{1}^{t}={\left[H_{1,1}=0,\cdots ,H_{5,1}=0\right]}^{T}=0$, where $H_{i,1}$ is the height of the *i*th object point at the first epoch. The shift of the object points between the measurement epochs is given by $\Delta X_{\left(1,2\right)}={\left[\Delta H_{1(1,2)},\cdots ,\Delta H_{5(1,2)}\right]}^{T}$, where $\Delta H_{i(1,2)}=H_{i,2}-H_{i,1}$. In the classical approach to the estimation of the point displacements, we used the functional model of Equation (1). Since all height differences were measured twice at two measurement epochs, namely, we had two series of measurements at each epoch, then we should assume that $y_{l}\in R^{32}$, $X_{l}={\left[H_{1,l},\cdots ,H_{5,l}\right]}^{T}$, and $A_{⊗}=A⊗1_{2}\in R^{32,5}$ where $A\in R^{16,5}$ is a known coefficient matrix related to one series of measurements, $1_{2}={\left[1,1\right]}^{T}$, and ⊗ is the Kronecker product. On the other hand, in the case of M_split_ estimation, we should apply the functional model of Equation (3) for which $y={\left[y_{1}^{T},y_{2}^{T}\right]}^{T}\in R^{64}$, $A={\left[A_{⊗}^{T},A_{⊗}^{T}\right]}^{T}\in R^{64,5}$, and $X_{\left(1\right)},X_{\left(2\right)}\in R^{5}$ are the competitive versions of the parameter vector, hence $v_{\left(1\right)},v_{\left(2\right)}\in R^{64}$ are the respective competitive versions of the measurement errors.

When analyzing the efficacy of M_split_ estimation, we can use two measures, namely the local measure of the distance between the LS and M_split_ estimates

(19) $$\begin{array}{l} \lambda_{(l)j}({\left[{\hat{X}}_{\left(l\right)}\right]}_{j},{\left[{\hat{X}}_{LS,l}\right]}_{j})=\\ \;=abs({\left[{\hat{X}}_{\left(l\right)}\right]}_{j}-{\left[X_{l}^{t}\right]}_{j})-abs({\left[{\hat{X}}_{LS,l}\right]}_{j}-{\left[X_{l}^{t}\right]}_{j}) \end{array}$$

as well as the global one

(20) $$\lambda_{\left(l\right)}({\hat{X}}_{\left(l\right)},{\hat{X}}_{LS,l})=\left\|{\hat{X}}_{\left(l\right)}-X_{l}^{t}\right\|-\left\|{\hat{X}}_{LS,l}-X_{l}^{t}\right\|$$

where ${\left[•\right]}_{j}$ is *j*th element of the vector and ‖•‖ is the Euclidean norm. The local distance, which is just another form of Equation (16), is related to a particular parameter, for example, the height of a displacing point. The global distance describes the whole parameter vector. Thus, we can define the local and global success rates in the following way

(21) $$\begin{array}{l} \gamma_{(l),j}({\left[{\hat{X}}_{\left(l\right)}\right]}_{j},{\left[{\hat{X}}_{LS,l}\right]}_{j};\Delta X_{\left(1,2\right)})=\frac{1}{N}\sum_{i=1}^{N}s_{(l),j}^{i}({\left[{\hat{X}}_{\left(l\right)}\right]}_{j},{\left[{\hat{X}}_{LS,l}\right]}_{j})\\ \gamma_{\left(l\right)}({\hat{X}}_{\left(l\right)},{\hat{X}}_{LS,l};\Delta X_{\left(1,2\right)})=\frac{1}{N}\sum_{i=1}^{N}s_{\left(l\right)}^{i}({\hat{X}}_{\left(l\right)},{\hat{X}}_{LS,l}) \end{array}$$

where $s_{(l)j}^{i}({\left[{\hat{X}}_{\left(l\right)}\right]}_{j},{\left[{\hat{X}}_{LS,l}\right]}_{j})$ and $s_{\left(l\right)}^{i}({\hat{X}}_{\left(l\right)},{\hat{X}}_{LS,l})$ are functions of an elementary success from Equation (17) and indexed with the respective arguments.

The empirical analysis, which was based on the MC method for N=5000 simulations, was carried out for several variants of the point displacements. First, we assumed that only point $P_{5}$ was displaced. The respective MC estimates obtained for the LS and M_split_ estimations and $\Delta H_{5(1,2)}=-50$, $\Delta H_{5(1,2)}=-100$, or $\Delta H_{5(1,2)}=-200$ mm are presented in Table 1, which also presents the local and global SRs.

**Figure 6:**
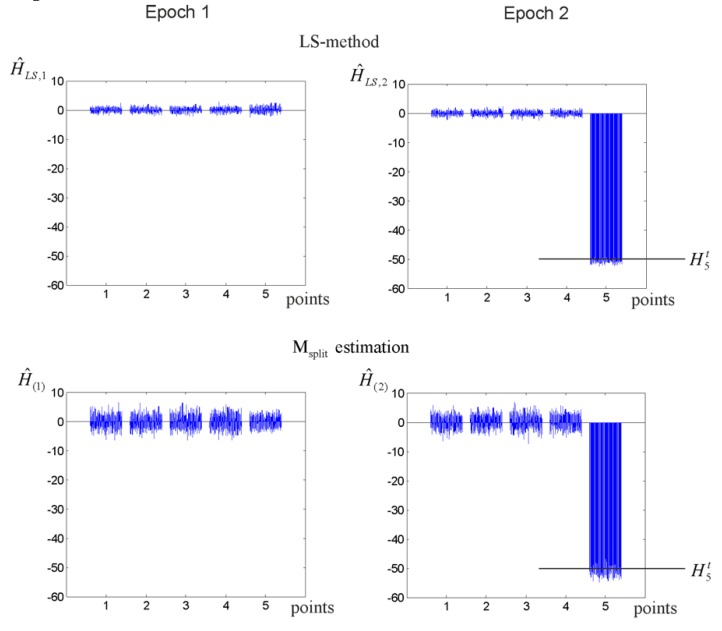
The LS and M_split_ estimates of the MC simulations ($\Delta H_{5(1,2)}=-50$ mm).

The MC estimates were similar for both estimation methods and the stable points. The SRs indicate that the LS estimates were closer to the theoretical values in the vast majority of the simulations. Note that the local SRs obtained for point $P_{5}$ were much higher than the global ones. All estimates of the point heights obtained in the MC simulations (for the variant $\Delta H_{5(1,2)}=-50$ mm) are presented in Figure 6.

**Figure 7:**
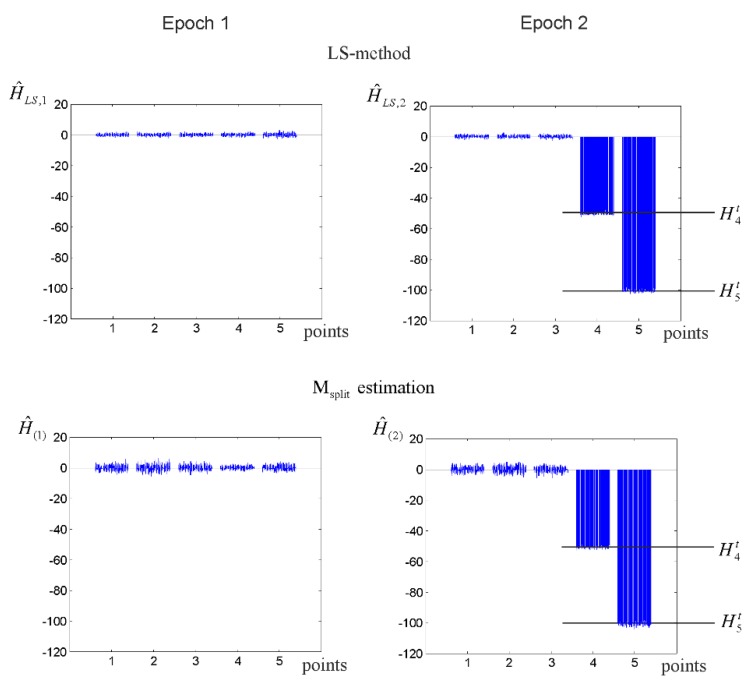
The LS and M_split_ estimates of the MC simulations ($\Delta H_{4(1,2)}=-50$ and $\Delta H_{5(1,2)}=-100$ mm).

In the second variant, we assumed that there were two unstable points, namely $P_{5}$ and $P_{4}$. The results, which were obtained for the different point shifts, are presented in Table 2. Here, the MC estimates obtained for both methods were also similar. Figure 7 presents the LS and M_split_ estimates that were obtained for all of the MC simulations. Generally, this confirmed the correctness of both estimation methods; however, differences between these two estimation methods were also apparent. The main difference was the dispersion, which was larger in the case of the M_split_ estimation, especially for the stable points, which suggests that the accuracy of the M_split_ estimation was worse than LS estimation. It is also worth noting that the SRs of the M_split_ estimation achieved bigger values in this variant. In the case of point $P_{5}$, the results of the M_split_ estimation were better than the results of the classical approach in almost one third of the simulations.

The results, which are presented here, show that both methods, namely LS and M_split_ estimation, yielded satisfactory solutions. However, such a conclusion was valid for the ordered observation sets, namely when each observation was properly assigned to its measurement epoch. If such a condition is not met, then the observation from another epoch will usually be regarded as an outlier. Since LS estimation as well as M_split_ estimation are not robust against outliers, they both break down (please note that M_split_ estimation is generally not robust unless we introduce an additional virtual model for outliers). Note that in the context addressed here, the outliers result from the assignment of an observation to the wrong measurement epoch, but not from gross errors. The natural feature of M_split_ estimation is the automatic assignment of each observation to the proper epoch. Thus, we can suppose that this estimation method will not break down if such outliers occur. To illustrate this feature of M_split_ estimation, we simulated that point $P_{5}$ was displaced and that $\Delta H_{5(1,2)}=-50$ mm. Now, let us consider the following variants of the observation sets: variant A, where both observation sets were correct (all observations were assigned to their epochs properly); variant B, where the observation $h_{16}$ at the second epoch was equal to $h_{16}$ at the first one, namely $h_{16}^{2}=h_{16}^{1}$; and variant C, where $h_{16}^{2}=h_{16}^{1}$, but also $h_{15}^{2}=h_{15}^{1}$. Thus, in variants B and C, we simulated that some observations that were assigned to the second measurement epoch should be related to the first one. The results obtained for all variants are presented in Table 3. In the case of variant A, the results were very close to the respective results presented in Table 1. If the observation sets are not ordered correctly, then the local SRs at the second epoch are close to 1, which means that almost always, the height of point $P_{5}$ at the second measurement epoch is better assessed by the M_split_ estimation than by LS estimation. Additionally, the global SRs were very high at the second epoch, hence one can say that the heights of all network points were better estimated by the application of M_split_ estimation.

## 4. Conclusions

The paper showed that M_split_ estimation can be successfully applied in deformation analysis. The results were generally similar to the results of the more conventional LS estimation; however, the latter method usually yielded slightly better outcomes. The elementary tests showed that the efficacy of the M_split_ estimation grew with an increasing shift between the observation sets. In the case of geodetic networks, where a parameter vector usually consists of several point coordinates, the shift of one or two such coordinates between measurement epochs does not influence the efficacy of the M_split_ estimation in a significant way. The real advantage of the M_split_ estimation was revealed for the disordered observation sets, for example, when the observations from at least two measurement epochs were mixed for some reason. Note that the LS estimates break down in such cases, in contrast with the M_split_ estimation, for which the ordering of all observations within the combined observation set can be arbitrary and does not influence the final results of the method as well as its iterative process. Such a feature results directly from the theoretical foundations of the method, which are based on the concept of the split potential. In short, each observation “chooses” the functional model that fits it best. In this context, M_split_ estimates are robust against some kind of “outliers”, namely observations that come from other observation sets. Referring to the presented example, there were four height differences regarding the height of network point $P_{5}$. If one of them does not fit the other, then the method tries to fit such an “outlying” observation into another epoch. If it works, then the whole estimation process succeeds. However, if such an observation is in fact affected by a gross error, then it does not fit any epoch, and the estimation must break down. The introduction of a virtual epoch, which is not related to any real measurements, is one solution to this problem. One can say that such an epoch can collect all “loners” that do not fit any real measurement epochs. Generally speaking, one can say that the M_split_ estimation is not robust against outliers, which results from the occurrence of gross errors. However, if one assumes an additional competitive functional model (dedicated to outliers), then the M_split_ estimation can estimate the location parameters for “good” observation aggregations as well as outlier(s). Increasing the number of competitive functional models protects the estimation of location parameters of good observations from the bad influence of outliers. Note that in this context, outliers are no longer “outlying”, and become regular observations of the third (or more generally next) aggregation. This concept, which is out of the scope of this paper, was discussed in [23,24,30].

## Figures and Tables

**Table 1:** The Monte Carlo estimates of the point heights and success rates for one unstable point.

$\Delta H_{5(1,2)}=-50$				$\Delta H_{5(1,2)}=-100$				$\Delta H_{5(1,2)}=-200$			
${\hat{X}}_{LS,1}$	${\hat{X}}_{\left(1\right)}$	${\hat{X}}_{LS,2}$	${\hat{X}}_{\left(2\right)}$	${\hat{X}}_{LS,1}$	${\hat{X}}_{\left(1\right)}$	${\hat{X}}_{LS,2}$	${\hat{X}}_{\left(2\right)}$	${\hat{X}}_{LS,1}$	${\hat{X}}_{\left(1\right)}$	${\hat{X}}_{LS,2}$	${\hat{X}}_{\left(2\right)}$
0.2	−3.1	0.4	2.9	−0.5	−1.1	−0.6	0.6	0.9	−1.8	−0.7	−0.4
1.4	−1.2	−1.0	0.9	−0.4	−1.3	0.7	2.9	0.5	0.7	0.5	−0.8
2.1	−0.6	−0.6	−0.6	0.1	−0.6	0.5	1.3	−0.3	−2.8	−0.1	1.1
-0.8	−3.6	−0.6	0.6	1.1	−0.9	−1.0	1.2	0.0	−0.4	−1.5	1.5
0.8	−1.9	−50.4	−49.1	0.3	−1.2	−99.8	−98.7	0.8	−0.8	−200.1	−199.7
$\gamma_{\left(1\right)}=0.018$ $\gamma_{(1),5}=0.172$		$\gamma_{\left(2\right)}=0.019$ $\gamma_{(2),5}=0.182$		$\gamma_{\left(1\right)}=0.020$ $\gamma_{(1),5}=0.177$		$\gamma_{\left(2\right)}=0.017$ $\gamma_{(2),5}=0.187$		$\gamma_{\left(1\right)}=0.025$ $\gamma_{(1),5}=0.171$		$\gamma_{\left(2\right)}=0.024$ $\gamma_{(2),5}=0.196$	

**Table 2:** The MC estimates of the point heights and SRs for two unstable points.

$\Delta H_{5(1,2)}=-50;\;\Delta H_{4(1,2)}=-50$				$\Delta H_{5(1,2)}=-100;\;\Delta H_{4(1,2)}=-50$				$\Delta H_{5(1,2)}=-200;\;\Delta H_{4(1,2)}=-50$			
${\hat{X}}_{LS,1}$	${\hat{X}}_{\left(1\right)}$	${\hat{X}}_{LS,2}$	${\hat{X}}_{\left(2\right)}$	${\hat{X}}_{LS,1}$	${\hat{X}}_{\left(1\right)}$	${\hat{X}}_{LS,2}$	${\hat{X}}_{\left(2\right)}$	${\hat{X}}_{LS,1}$	${\hat{X}}_{\left(1\right)}$	${\hat{X}}_{LS,2}$	${\hat{X}}_{\left(2\right)}$
−0.2	−0.5	−0.3	0.6	−0.5	0.1	−0.4	0.3	0.0	−1.3	−0.3	−1.1
−0.4	−2.0	−0.1	−0.1	0.2	0.5	−0.2	−0.4	0.0	−0.2	−0.1	0.9
−0.4	−0.4	−0.9	0.2	0.1	−0.3	0.2	−0.3	−0.2	−0.2	−0.3	−0.4
−0.1	−0.3	−50.5	−50.1	0.4	0.5	−49.9	−49.6	−0.1	−0.4	−50.0	−50.1
−0.5	−1.4	−50.1	−50.2	−0.6	−0.4	−100.1	−99.8	−0.5	−0.8	−200.3	−200.2
$\gamma_{\left(1\right)}=0.070$ $\gamma_{(1),5}=0.272$		$\gamma_{\left(2\right)}=0.070$ $\gamma_{(2),5}=0.268$		$\gamma_{\left(1\right)}=0.080$ $\gamma_{(1),5}=0.281$		$\gamma_{\left(2\right)}=0.080$ $\gamma_{(2),5}=0.288$		$\gamma_{\left(1\right)}=0.103$ $\gamma_{(1),5}=0.314$		$\gamma_{\left(2\right)}=0.105$ $\gamma_{(2),5}=0.312$	

**Table 3:** The MC estimates of the point heights and SRs for the disturbed observation sets.

Variant A: Correct Order				$\mathrm{Variant}\;B:\;h_{16}^{2}=h_{16}^{1}$				$\mathrm{Variant}\;C:\;h_{15}^{2}=h_{15}^{1}$ $,\;h_{16}^{2}=h_{16}^{1}$			
${\hat{X}}_{LS,1}$	${\hat{X}}_{\left(1\right)}$	${\hat{X}}_{LS,2}$	${\hat{X}}_{\left(2\right)}$	${\hat{X}}_{LS,1}$	${\hat{X}}_{\left(1\right)}$	${\hat{X}}_{LS,2}$	${\hat{X}}_{\left(2\right)}$	${\hat{X}}_{LS,1}$	${\hat{X}}_{\left(1\right)}$	${\hat{X}}_{LS,2}$	${\hat{X}}_{\left(2\right)}$
0.0	2.2	0.3	−1.1	0.4	−1.5	−6.8	0.3	−0.8	0.4	−4.5	−5.2
0.4	−0.1	1.1	0.4	−0.5	−1.5	2.1	1.8	−0.2	−0.8	−5.3	−7.7
0.6	0.8	0.3	−1.5	−0.6	−3.6	3.4	1.6	−0.1	−1.0	4.9	7.4
−0.7	−0.9	0.0	1.0	0.3	−1.4	2.0	2.4	−1.3	−0.6	5.2	7.1
−0.2	0.5	−49.8	−50.3	0.4	−1.5	−36.2	−46.5	−2.0	−1.0	25.3	−42.6
$\gamma_{\left(1\right)}=0.018$ $\gamma_{(1),5}=0.172$		$\gamma_{\left(2\right)}=0.019$ $\gamma_{(2),5}=0.210$		$\gamma_{\left(1\right)}=0.127$ $\gamma_{(1),5}=0.321$		$\gamma_{\left(2\right)}=0.875$ $\gamma_{(2),5}=0.986$		$\gamma_{\left(1\right)}=0.263$ $\gamma_{(1),5}=0.474$		$\gamma_{\left(2\right)}=0.887$ $\gamma_{(2),5}=0.998$	
